# Amyloid Goiter Associated with Amyloidosis Secondary to Rheumatoid Arthritis

**DOI:** 10.1155/2013/792413

**Published:** 2013-12-04

**Authors:** Gungor Uzum, Fatih Oner Kaya, Ayse Kubat Uzum, Meltem Kucukyilmaz, Mehmet Emin Gunes, Yigit Duzkoylu, Cem Leblebici, Oguz Koc, Yavuz Selim Sari

**Affiliations:** ^1^Department of General Surgery, Istanbul Education and Research Hospital, Turkey; ^2^Department of Internal Medicine, Istanbul Education and Research Hospital, Turkey; ^3^Division of Endocrinology and Metabolism, Department of Internal Medicine, Istanbul Faculty of Medicine, Istanbul University, Turkey; ^4^Department of Pathology, Istanbul Education and Research Hospital, Turkey

## Abstract

Amyloidosis refers to a variety of conditions in which amyloid proteins are abnormally deposited in organs and/or tissues. The most common forms of systemic amyloidosis are primary amyloidosis (PA) of light chains and secondary amyloidosis (SA) caused by chronic inflammatory diseases such as rheumatoid arthritis (RA). Although involvement of the thyroid gland by amyloid is a relatively common phenomenon, clinically significant enlargement of the thyroid owing to amyloid deposition is a rare occurrence. In SA, the deposition of amyloid associated (AA) protein is associated with atrophy of thyroid follicles. The clinical picture of these patients is characterized by rapid, painless thyroid gland enlargement which may be associated with dysphagia, dyspnea, or hoarseness. Thyroid function is not impaired in most cases. Although amyloid goitre secondary to systemic amyloidosis due to chronic inflammatory diseases is relatively common, specifically related to RA is much more uncommon one and it is reported less in the literature. In this report, A 52-old-year female patient with amyloid goiter associated with amyloidosis secondary to rheumatoid arthritis is presented.

## 1. Introduction

Amyloidosis refers to a variety of conditions in which amyloid proteins are abnormally deposited in organs and/or tissues. Primary amyloidosis (PA) arises from a disease with disordered immune cell function such as multiple myeloma and other immunocyte dyscrasias. Secondary (reactive) amyloidoses (SA) are those occurring as a complication of some other chronic inflammatory or tissue destructive diseases. Rheumatoid arthritis (RA) is an autoimmune disease that results in a chronic, systemic inflammatory disorder that may affect many tissues and organs.

The most common forms of systemic amyloidosis are PA of light chains and SA caused by chronic inflammatory diseases such as RA [1–4]. James first reported the presence of clinically detectable thyroid enlargement because of amyloid deposition in 1858, followed by Eiselberg in 1904, who later coined the term “amyloid goitre” [5]. Although involvement of the thyroid gland by amyloid is a relatively common phenomenon, clinically significant enlargement of the thyroid owing to amyloid deposition is an extremely rare occurrence [6–8]. It affects the thyroid in a bilateral and diffuse manner. Preoperatively it simulates a multinodular goitre and surgical intervention is often necessary to establish a diagnosis and to relieve compressive symptoms of a neck mass. Here, in this case report, a 52-old-year female patient with amyloid goiter associated with amyloidosis secondary to RA is presented.

## 2. Case Report 

**Figure 1:**
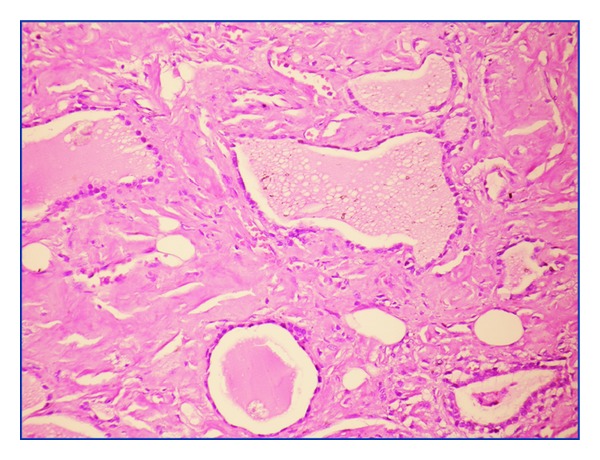
Expansive amorphous eosinophilic substance deposition and lipid infiltration in the whole thyroid gland (HE ×200).

**Figure 2:**
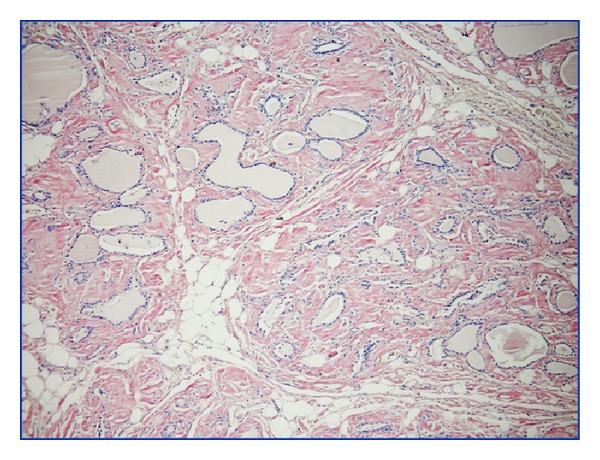
Amyloid deposition stained red in the interstitial space and apple-green birefringence with the polarizing microscope (Kongo ×100).

The patient was a 52-year-old woman that is referred to the general surgery clinic presenting with increasing dyspnea and a visible swelling at the base of the neck for the last two years. She had been diagnosed with RA six years ago but had not used the prescribed medication regularly. The thyroid gland was rough in palpation. In laboratory data, free T4: 1.26 ng/dL (N: 0.82–1.77 ng/dL), TSH: 0.243 mIU/mL (N: 0.27–4.2 mIU/mL), and thyroid autoantibodies such as Anti-TPO (antithyroid peroxidase) and anti-Tg (antithyroglobulin) were found to be negative. Other laboratory findings were WBC: 8000, Hb: 9.6 g/DL, Hct: 29.4%, MCV: 89.3 fL, Plt: 216000 *μ*L, urea: 89 md/dL, and creatinine: 2.2 mg/dL, and hepatic function tests were found to be normal. Both of the thyroid lobes were enlarged in the ultrasound examination. In the right lobe, we found smooth, multiple nodules of 45–50 mm size that contained cystic degeneration areas. Biopsy specimens driven by fine needle aspiration did not show any malignant findings. Renal ultrasound examination revealed grade II nephropathy. Total thyroidectomy was performed because of the evident pressure symptoms. Thyroid tissue was found to be very rough and the histopathological examination revealed that macroscopically the right lobe was 12 × 6 × 5 cm in size and contained four nodules of 45–50 mm size. The size of the left lobe was 10 × 6 × 5 cm and the whole material was in pale grey-brown colour. The resected material was stained with haematoxylin-eosin dye and revealed an expansive amorphous eosinophilic substance deposition and lipid infiltration in the whole thyroid gland (Figure 1). An adenomatous hyperplasia was also found in the right lobe. On Congo red dye staining, amyloid deposition was stained red in the interstitial space and it showed an apple-green birefringence with the polarizing microscope (Figure 2). Amyloid deposition was sensitive to alkaline (potassium permanganate), causing fading of the stain. In the postoperative 16th month, chronic renal failure occurred and haemodialysis treatment was started. Nine months later, the patient died of myocardial infarction.

## 3. Discussion

The preoperative diagnosis of amyloid goiter should be considered in patients with known systemic amyloidosis or with a long-standing predisposing disease who present a rapidly growing thyroid volume in association with an euthyroid state.

In our patient, amyloid goiter probability had been considered but because of the evident pressure symptoms, after FNA examination, surgery was performed rather than diagnosing a systemic amyloidosis preoperatively. In the literature, there are findings about the decreasing in the symptoms and amyloid goiter after treatment of the primary disease [9].

In autopsy studies, amyloid material is found in the thyroid gland of 80% of patients with SA and 50% of patients with PA. In a study, a systematic review of the published data on amyloid goiter was carried out for the period from 1951 to March 2008. A total of 127 publications (case series, single-case reports, and reviews) were found, of which 31 were case series published from February 1995 to March 2008 [10]. Although involvement of the thyroid gland by amyloid is a relatively common phenomenon, clinically significant enlargement of the thyroid owing to amyloid deposition is a rare occurrence. Although it is very rare, in some cases isolated thyroid amyloidosis may be seen as chronic inflammatory diseases [10]. In the present case, high levels of urea and creatinine preoperatively and the necessity of haemodialysis postoperatively because of chronic renal failure make us think that the amyloidosis was systemic in the patient. Unfortunately, we had not performed renal biopsy.

In every patient with RA who is presenting with symptoms of goitre, amyloid goitre must be taken into consideration. In addition, amyloid goiter, specifically secondary to RA, is extremely uncommon in the literature [11–14]. We presented a patient of amyloid goitre having RA with the recent literature.
